# Pure high-grade fetal adenocarcinoma of the lung: a case report

**DOI:** 10.1186/s40792-018-0478-4

**Published:** 2018-07-04

**Authors:** Yasuaki Kubouchi, Yuki Matsuoka, Kunio Araki, Yoshiteru Kidokoro, Tomohiro Haruki, Hiroshige Nakamura, Yoshihisa Umekita

**Affiliations:** 1 Division of Organ Pathology, Department of Pathology, Faculty of Medicine, Tottori University, Tottori, Japan; 2 Division of General Thoracic Surgery, Department of Surgery, Tottori University, 86, Nishicho, Yonago, Tottori 683-8503 Japan; 3 Department of Surgery, National Hospital Organization Matsue Medical Center, Shimane, Japan; 4 Department of Surgery, Tottori Prefectural Kosei Hospital, Tottori, Japan

**Keywords:** H-FLAC, Adenocarcinoma, Lung cancer

## Abstract

**Background:**

Pure high-grade fetal adenocarcinoma of the lung (H-FLAC) is a very rare tumor.

**Case presentation:**

An annual check-up revealed an abnormal shadow in the left middle lung field of a 63-year-old Japanese man. Chest computed tomography (CT) showed a 3.6 × 2.8 cm pulmonary lesion with clear boundary in the left upper lobe. A transbronchial lung biopsy revealed non-small cell carcinoma. A left upper lobectomy and mediastinal lymph node dissection were performed. Histologically, the tumor consisted of solid proliferation of atypical cell with clear cytoplasm. Another histological component and morulae were not contained. Immunohistochemically, the tumor was focally positive for alpha-fetoprotein (AFP) and beta-catenin in the cell membrane. Accordingly, we made the diagnosis of pure H-FLAC, pT2aN2M0, stage IIIA. Two courses of adjuvant chemotherapy (cisplatin and vinorelbine) were administered but then the treatment was discontinued due to the patient’s adverse reaction. At 25 months after the surgery, the patient had relapsed.

**Conclusions:**

We report a very rare case of pure H-FLAC. This histology has been considered to predict an extremely poor prognosis; therefore, the elucidation of genetic abnormalities and effective treatment is awaited.

## Background

Fetal lung adenocarcinoma, which was named in 2011 in accord with the International Association for the Study of Lung Cancer, the American Thoracic Society and the European Respiratory Society (IASLC/ATS/ERS) classification of lung adenocarcinoma in 2011 [1], was first reported as ‘pulmonary endodermal tumor resembling fetal lung’ in 1982 [2]. Fetal lung adenocarcinoma is further classified as low-grade fetal adenocarcinoma (L-FLAC) and high-grade fetal adenocarcinoma (H-FLAC) [1], and H-FLAC is an especially rare tumor that is thought to be expressed as clear cell adenocarcinoma with fetal lung features or high-grade lung adenocarcinoma with fetal lung-like morphology [3, 4].

Most H-FLACs are mixed H-FLAC, combined with conventional-type adenocarcinoma (acinar, papillary, and lepidic), large cell neuroendocrine carcinoma (LCNEC), or another type of carcinoma [5]. To the best of our knowledge, only five cases of pure H-FLAC have been reported [5, 6]. Here, we present the sixth reported case of pure H-FLAC.

## Case presentation

**Fig. 1:**
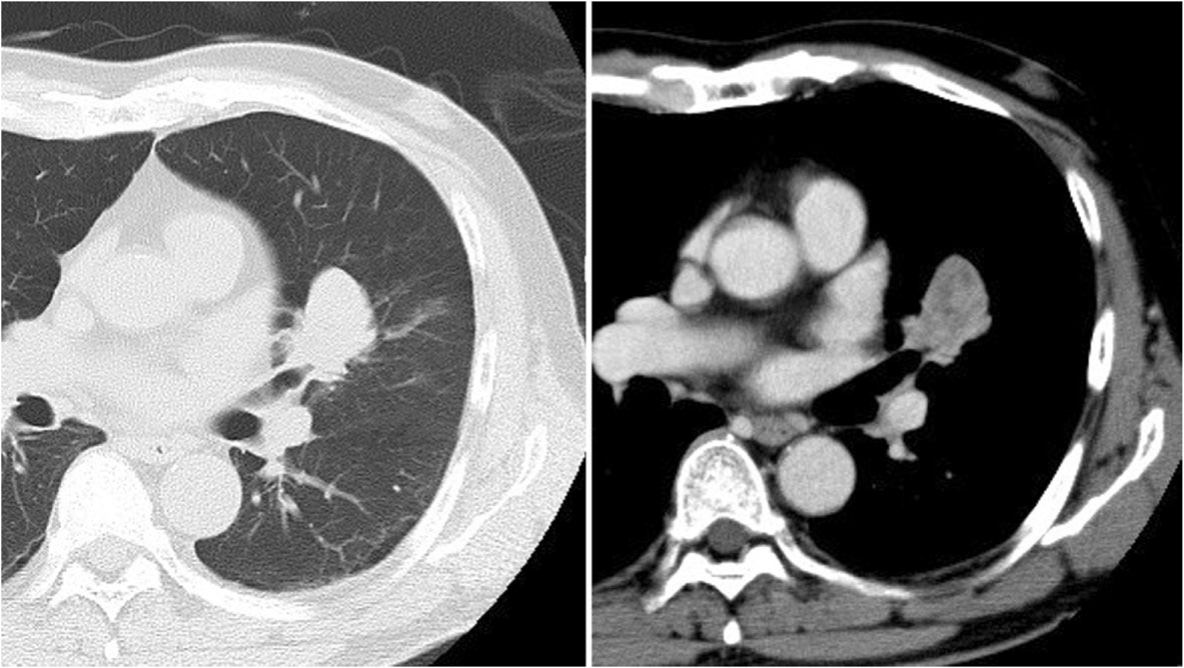
A 3.6 × 2.8 cm mass with a clear boundary and heterogeneous contrast enhancement was present in the left upper lobe on chest CT

A 63-year-old Japanese man, a current smoker of more than 45 pack-years, was revealed by routine examination’s chest X-ray to have an abnormal shadow in the left middle lung field. His past medical history was unremarkable. A chest computed tomography (CT) scan showed a 3.6 × 2.8 cm pulmonary lesion in the left upper lobe, with a clear boundary and heterogeneous contrast enhancement (Fig. 1). Positron emission tomography (PET) showed an accumulation of 18F-fluorodexyglucose (FDG) with a maximum standardized uptake value (SUV max) of 4.95 in the early phase and 6.31 in the late phase in the nodule. No accumulation of FDG was noted in the pulmonary hilum, or mediastinal lymph nodes.

A trans-bronchial lung biopsy (TBLB) of the mass in the left upper lobe was performed; it revealed non-small cell carcinoma. Although the serum carcinoembryonic antigen (CEA) level was 52.1 ng/mL (normal range 0–5.0 ng/mL), there were no distant metastases or other cancer lesions. The preoperative clinical diagnosis of primary lung cancer, stage cT2aN0M0 stage IB was considered.

A left upper lobectomy and mediastinal lymph node dissection were performed by video-assisted thoracic surgery. The resected tumor measured 3.8 × 2.8 × 2.8 cm in diameter.

**Fig. 2:**
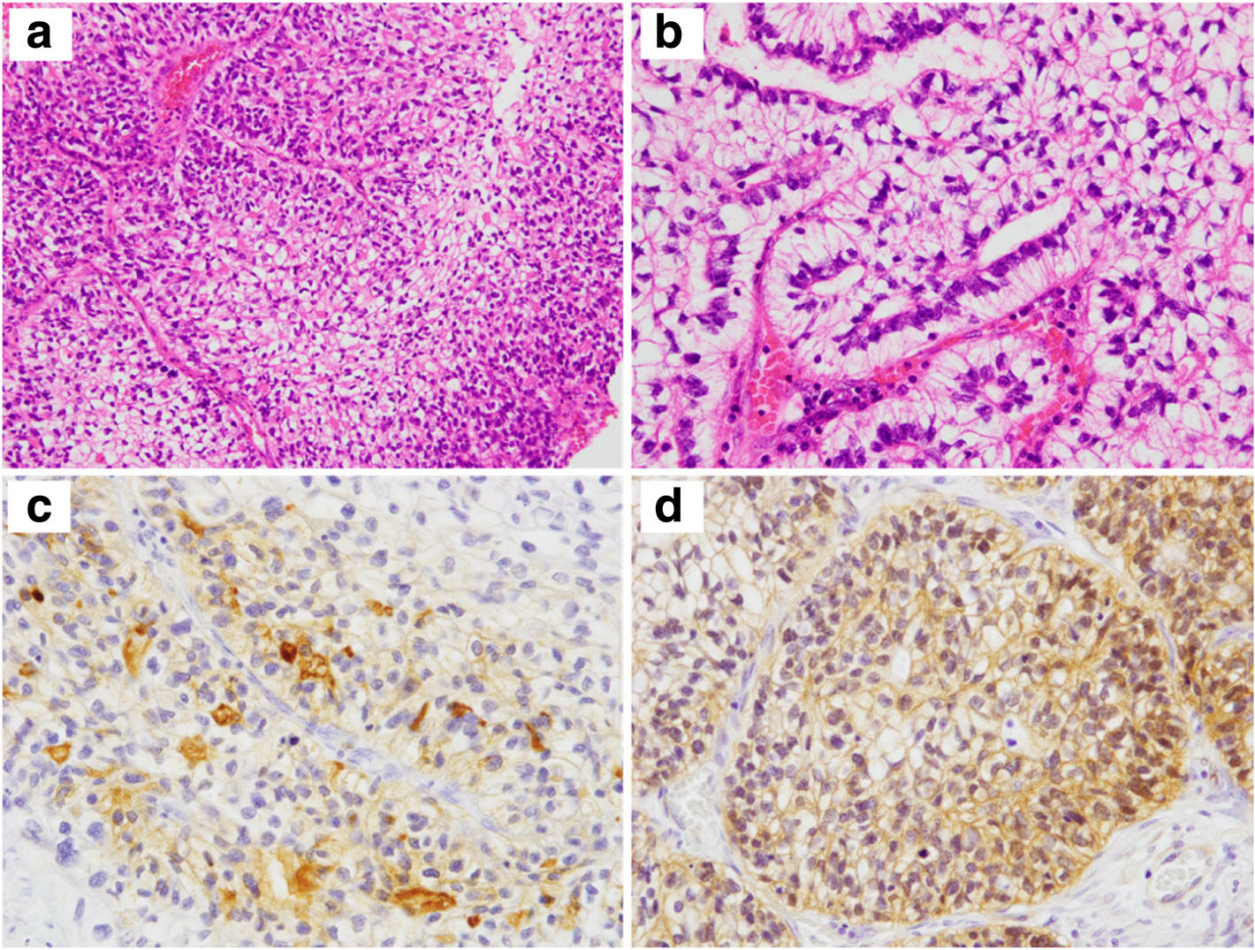
**a**, **b** On the histopathological examination, atypical cells with a columnar clear cell pattern proliferated in a sheet pattern and complex glandular structures. **c** Immunohistochemically, the tumor cells were focal positive for AFP. **d** Beta-catenin was positive in the cell membrane

Grossly, the specimen showed a well-established boundary as a grayish lesion with areas of necrosis. The histopathological examination showed a solid proliferation of columnar atypical cells with cell cytoplasm and complex glandular structures with abundant desmoplastic stroma, a morphological resemblance to fetal lung and significant tumor necrosis (Fig. 2a, b). Conventional lung adenocarcinoma, another histological component and morulae were not found. Periodic acid Schiff (PAS) staining demonstrated glycogen in the cytoplasm of the neoplastic cells. Stains for mucin were negative. The immunohistochemical analysis showed positivity for AE1/AE3, CEA, and alpha-fetoprotein (AFP) (focal) (Fig. 2c). Thyroid transcription factor-1 (TTF-1), chromogranin A, synaptophysin, CD56, and p40 were negative. The beta-catenin result was positive, predominantly in the cell membrane (Fig. 2d). The final histopathological diagnosis was H-FLAC.

Both lymphatic and vascular invasion were detected. Microscopically, all margins were free and mediastinal lymph nodes (#5) showed metastasis. The final pathological staging was stage IIIA (pT2aN2M0). Both epidermal growth factor receptor (EGFR) mutations and anaplastic lymphoma kinase (ALK) gene rearrangements were negative. The expression of programmed cell death 1 ligand 1 (PD-L1) (Dako, clone 22C3) by resected specimen was negative.

The patient received cisplatin plus vinorelbine as adjuvant chemotherapy, but it was canceled at two courses because of the dehydration and hyperglycemia induced by the chemotherapy. At 25 months after the surgery, the patient had relapsed pleural dissemination. Postoperative serum CEA and AFP was 1.5 and 3.7 ng/mL, respectively. But, serum CEA at the time of relapse was 132.4 ng/mL, and AFP was unfortunately not searched.

## Discussion

Fetal adenocarcinoma is subcategorized into L-FLAC and H-FLAC by the IASLC/ATS/ERS classification of lung adenocarcinoma based on differences in clinicopathological features and prognoses. It was reported that H-FLAC constituted 0.4% of all primary lung cancer cases [4], and 0.54% of primary lung adenocarcinomas [6]. H-FLAC has been reported to be more common in men, and almost all H-FLAC patients smoke or smoked; symptoms such as cough and blood sputum were observed in approx. one-half of patients [3], but our patient showed no symptoms.

It was reported that approx. 40% of H-FLAC case were discovered at stage II or higher, and approx. 40% of the patients died within 2 years after surgery [3]. Several studies reported that H-FLAC might be termed ‘pulmonary clear cell carcinoma with fetal lung features’ [3, 7], and 80–100% of H-FLAC cases were combined with a conventional-type adenocarcinoma, a large cell neuroendocrine carcinoma, or an enteric adenocarcinoma [4–6]. A type in which histological types other than H-FLAC are mixed in tumor lesion is referred to as mixed H-FLAC, whereas entire tumor lesions is H-FLAC is called pure H-FLAC. In this case, the resected specimen was sliced at 5-mm intervals and the entire tumor was microscopically searched, but no lesion other than H-FLAC was found. Pure H-FLAC is a very rare tumor, and the clinicopathological features of pure H-FLAC are not yet established.

We were able to identify only five reported cases of pure H-FLAC from several institutions [5, 6], and the six cases (including the present case) are summarized in Table 1. The mean age of the patients was 69.2 years, and almost all of the patients had a smoking history. The mean tumor size was 63.7 mm. Suzuki et al. reported that the mean age and tumor size of mixed H-FLAC cases were 66.1 years and 29.3 mm, respectively [5]. Compared with those cases, pure H-FLAC tends to be more common in the elderly and to be of larger size.

**Table 1:** Summary of pure high-grade fetal adenocarcinoma of the lung

Study	No.	Age	Sex	Smoking history	Tumor size (mm)	p-Stage	TTF-1	AFP	Pleural invasion	LN metastasis	EGFR	ALK	Follow-up (months)
Suzuki et al. [5]	1	67	M	+	55	IV	–	+	–	–	–	–	DOD (7)
Suzuki et al. [5]	2	73	M	NA	34	IIA	–	+	–	+	–	–	PD
Suzuki et al. [5]	3	67	M	+	115	IIB	–	+	–	–	–	–	AWD 11
Suzuki et al. [5]	4	81	M	+	50	IIB	–	+	–	–	–	–	NA
Zhang et al. [6]	5	64	M	+	90	IIIB	–	+	+	+	–	–	DOD (9)
Present case, 2018	6	63	M	+	38	IIIA	–	+	+	+	–	–	AWD (25)

*DOD* died with disease, *PD* perioperative death, *AWD* alive with disease, *NA* data not available

The prognosis of pure H-FLAC has been very poor. As seen in Table 1, all six of the cases were stage II or more and most cases had relapsed within 2 years. On the other hand, only 13 out of 37 cases of previously reported mixed H-FLAC experienced relapse [4–6]. Although there are few cases and the observation period is short, pure H-FLAC is considered to have poor prognosis. Thus, it is important to distinguish between pure and mixed H-FLAC. Immunohistochemically, all cases were negative for TTF-1, whereas beta-catenin was positive in the cell membrane. Suzuki et al. revealed that there were no significant histological or immunohistochemical differences between the pure and mixed types of H-FLAC, although the pure type tended to show more tightly packed and complex glandular structures and no immunoexpression of TTF-1 [5]. In that report, 63% of the mixed H-FLACs were positive for TTF-1 [5]. No mutations were found in EGFR or ALK. Regarding the expression of PD-L1, there was no report mentioned so far, it is necessary to accumulate and consider cases in the future.

## Conclusions

We report a very rare case of pure H-FLAC. This histology has been considered to predict an extremely poor prognosis; therefore, the elucidation of genetic abnormalities and effective treatment is awaited.

## Abbreviations

AFP: Alpha-fetoprotein
ALK: Anaplastic lymphoma kinase
CEA: Carcinoembryonic antigen
CT: Computed tomography
EGFR: Epidermal growth factor receptor
FDG: Fluorodexyglucose
H-FLAC: High-grade fetal adenocarcinoma
LCNEC: Large cell neuroendocrine carcinoma
L-FLAC: Low-grade fetal adenocarcinoma
PAS: Periodic acid Schiff
PD-L1: Programmed cell death 1 ligand 1
PET: Positron emission tomography
SUV max: Maximum standardized uptake value
TBLB: Trans-bronchial lung biopsy
TTF-1: Thyroid transcription factor-1
